# Novel biomarkers with promising benefits for diagnosis of cervical neoplasia: a systematic review

**DOI:** 10.1186/s13027-020-00335-2

**Published:** 2020-11-16

**Authors:** Calleb George Onyango, Lilian Ogonda, Bernard Guyah, Clement Shiluli, Gregory Ganda, Omenge Elkanah Orang’o, Kirtika Patel

**Affiliations:** 1 Department of Biomedical Sciences and Technology, Maseno University, P.O Box Private Bag, Maseno, Kenya; 2 Department of Clinical Services, Division of Gynecology / Oncology, Jaramogi Oginga Odinga Teaching and Referral Hospital (JOOTRH), P.O Box 849, Kisumu, Kenya; 3 Department of Reproductive Health, Division of Gynecology / Oncology, Moi University, P. O Box 4606, Eldoret, Kenya; 4 Department of Immunology, Moi University, P.O Box 4606, Eldoret, Kenya

**Keywords:** MicroRNA, p16INKa / ki-67, HPV E6/E7/mRNA, DNA methylation, SCC-Ag. Performance

## Abstract

**Background:**

Cervical cancer screening is slowly transitioning from Pappanicolaou cytologic screening to primary Visual Inspection with Acetic Acid (VIA) or HPV testing as an effort to enhance early detection and treatment. However, an effective triage tests needed to decide who among the VIA or HPV positive women should receive further diagnostic evaluation to avoid unnecessary colposcopy referrals is still lacking. Evidence from experimental studies have shown potential usefulness of Squamous Cell Carcinoma Antigen (SCC Ag), Macrophage Colony Stimulating Factor (M-CSF), Vascular Endothelial Growth Factor (VEGF), MicroRNA, p16INKa / ki-67, HPV E6/E7/mRNA, and DNA methylation biomarkers in detecting premalignant cervical neoplasia. Given the variation in performance, and scanty review studies in this field, this systematic review described the diagnostic performance of some selected assays to detect high-grade cervical intraepithelial neoplasia (CIN2+) with histology as gold standard.

**Methods:**

We systematically searched articles published in English between 2012 and 2020 using key words from PubMed/Medline and SCOPUS with two reviewers assessing study eligibility, and risk of bias. We performed a descriptive presentation of the performance of each of the selected assays for the detection of CIN2 + .

**Results:**

Out of 298 citations retrieved, 58 articles were included. Participants with cervical histology yielded CIN2+ proportion range of 13.7–88.4%. The diagnostic performance of the assays to detect CIN2+ was; 1) SCC-Ag: range sensitivity of 78.6–81.2%, specificity 74–100%. 2) M-CSF: sensitivity of 68–87.7%, specificity 64.7–94% 3) VEGF: sensitivity of 56–83.5%, specificity 74.6–96%. 4) MicroRNA: sensitivity of 52.9–67.3%, specificity 76.4–94.4%. 5) p16INKa / ki-67: sensitivity of 50–100%, specificity 39–90.4%. 6) HPV E6/E7/mRNA: sensitivity of 65–100%, specificity 42.7–90.2%, and 7) DNA methylation: sensitivity of 59.7–92.9%, specificity 67–98%.

**Conclusion:**

Overall, the reported test performance and the receiving operating characteristics curves implies that implementation of p16ink4a/ki-67 assay as a triage for HPV positive women to be used at one visit with subsequent cryotherapy treatment is feasible. For the rest of assays, more robust clinical translation studies with larger consecutive cohorts of women participants is recommended.

**Supplementary Information:**

The online version contains supplementary material available at 10.1186/s13027-020-00335-2.

## Background

Cervical cancer cases continue to rise despite concerted efforts to provide rapid and effective screening coupled with intensified human papilloma virus (HPV) vaccination to selected age category of females [1]. Currently, the global incidence and mortality estimate of cervical cancer have risen to 569,847 and 311,365 respectively, with cases in sub-Saharan countries such as Kenya reported at 5250 (12.9%) and 3286 (11.84%) annually respectively, majorly from infection with high risk human papilloma virus (HR-HPV)16/18 [1].. Cervical carcinogenesis is characterized majorly by 1) increased expression of E6 and E7 genes of high risk HPVs, known to bind to and inactivate p53 and pRb oncosuppressors; 2) integration of viral DNA into host genome, with disruption of E2 viral genes and host chromosomal loci; and 3) molecular alterations of key regulators of cell cycle; all of which can be examined to predict a possible neoplasia using suitable probes such as DNA, RNA, antibody, protein, and aptamers [2, 3]. Today, great efforts have been made to identify novel biomarkers aiming to improve detection of the invasive cervical cancer at the earliest stage possible. This review examined, and highlighted some of the substantially tested biomarkers with promising diagnostic potential for premalignant cervical lesion, and the feasibility of their implementation as alternative triage tests for visual inspection with acetic acid (VIA) or HPV-DNA positive women in facilities with inadequate histology infrastructure.

Recent discoveries have demonstrated significant milestones in management of cervical cancer based on United States of America Food and Drug Administration (USFDA) approved biomarkers such as Squamous Cell Carcinoma Antigen (SCC-Ag) currently implemented to support physicians with rapid screening of women at high risk of cervical neoplasia, and complemented with Cancer Antigen 125 (CA-125), Serum Fragment of Cytokeratins (CYFRA), Soluble CD44 (sCD44), and Carcinoma Embryonic Antigen (CEA) as prognostic markers for pre-treatment prediction and disease monitoring [4]. Subsequently, new biomarkers such as Macrophage Colony Stimulating Factor (M-CSF), Vascular Endothelial Growth Factor (VEGF), MicroRNA, p16INKa / ki-67, HPV E6/E7/mRNA and DNA methylation have equally been identified in the recent past as potentially useful for early detection of cervical neoplasia [5–8]. The aim of this systematic review was to describe the diagnostic performance of some selected biomarkers to detect high-grade cervical lesions (CIN2+) with histology as gold standard; and evaluated their implementation feasibility based on investigators findings, remarks and applicability as triage test for VIA or HPV positive women in low income settings.

## Methods

### Protocol registration

In accordance to Maseno University study guidelines, our systematic review protocol was submitted to the Maseno University Register for study protocols Ref. No.PG/PHD/PH/00086/2017, and to the Open Science Framework (OSF) Register of Systematic Reviews.

### Eligibility criteria

The study design was based on selected studies: we considered cross-sectional and cohort studies that reported the diagnostic performance of SCC Ag, M-CSF, VEGF, miRNA (miR-9), p16INKa / ki-67, HPV E6/E7 mRNA and DNA methylation (majorly EPB41L3, JAM3, SOX1, L1) for the detection of CIN2+ (CIN2+ refers to: histologically confirmed high-grade lesions (CIN2, CIN3 and cancer)). We included studies of women with different kinds of cervical pathology published in peer-reviewed English journal articles in the period of 2012 to 2020 with the outcome of interest reported in different countries.

### Information sources and search strategy

This review was done following the Preferred Reporting Items for Systematic Reviews and Meta-Analysis Protocol (PRISMA) guidelines [9]. Research papers were systematically searched in PubMed/Medline and SCOPUS using key words by combing using Boolean operator. Additionally, manual search from Google scholar and Google databases was performed for grey literature, with last search done on 10th September 2020. The reference lists of retrieved articles were probed (forward and back ward searching) to identify articles that were not retrieved from databases and our manual search. The first two authors; C.G.O and L.O., searched the articles independently. The domains of the search terms were HPV E6/E7 mRNA, miR-9, p16INK4a / ki-67, DNA methylation, Squamous Cell carcinoma Antigen (SCC-Ag), Macrophage Colony Stimulating Factor (M-CSF), Vascular Endothelial Growth Factor (VEGF), DNA methylation, and Cervical Intraepithelial Neoplasia. We combined each of the studied biomarkers with the Boolean operator “OR”, and the result was combined with the other terms.

### Study selection

Research papers that reported the type of miRNA (miR-9), p16INK4a / ki-67, DNA methylation, SCC-Ag, M-CSF, VEGF, and HPV E6/E7 mRNA diagnostic performance for the detection of CIN2+ were included. Searched articles were directly imported and handled using EndNote X5 citation manager (Thomson Reuters, New York, USA). Based on the PRISMA protocol included in Additional file 1, duplicated articles were excluded, and the titles and abstracts of the remaining papers were screened independently for inclusion in full text evaluation by the first two authors.

### Data collection process and data items

Data such as the name of the first author, year of publication, country where the study was conducted, CIN profile of the study participants, sample type, the proportion of CIN2+, type of diagnostic test for each category of biomarkers, mean turnaround time (TAT), approval status of each assay, the positivity rate of each diagnostic assay, and its diagnostic performance (in terms of sensitivity, specificity, Positive Predictive value (PPV) and Negative Predictive Value (NPV)) were extracted from the included articles.

### Quality appraisal

To assess the risk of bias, the Critical Appraisal Skills Programme (CASP) tool [10], that was developed to evaluate studies of diagnostic test accuracy was independently used by the first two authors. Of the eleven criterion of the tool, we eliminated three items because their scoring was difficult. Assessment of quality results was categorized but not summarized into a score since the method has less validity [10].

### Data synthesis

The extracted data were fed into a Microsoft Excel and presented in terms of 1) CIN profile of the study subjects, 2) the proportion of miR-9, p16INK4a / ki-67, DNA methylation, SCC-Ag, M-CSF, VEGF and HPV E6/E7 test result 3) diagnostic performance of each of the seven assays to detect CIN2+ (sensitivity, specificity, PPV and NPV). We performed a descriptive presentation of these elements to compile a best evidence synthesis for the listed assays in the detection of CIN2+. A systematic narrative synthesis was provided in which summary results of recent studies with performance indicators were presented using text, figure, and table. Descriptive statistics, such as percentages were used to describe the findings.

## Results

### Search results

**Fig. 1:**
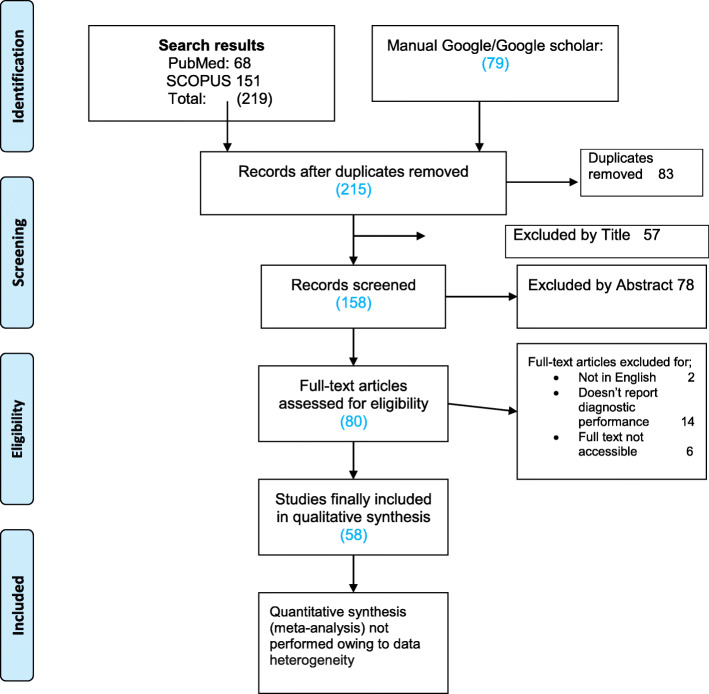
PRISMA flow diagram of literature selection

From the systematically searched databases and other sources, a total of 298 articles were retrieved and sequentially screened. After removing 83 duplicates, the 215 were further screened by title then 57 were removed. Additionally, 78 were removed by abstract and 22 removed by full text with justifiable reasons described in the PRISMA flow chart (Fig. 1) adopted from PRISMA guidelines for systematic screening [9]. Finally, a total of 58 studies met our inclusion criteria.

### Characteristics of the included studies

The characteristics of articles with performance indicators reported from different countries in Europe, Asia and the United States of America are summarized in (Table 1). We didn’t find articles reported in Latin America and Africa. The number of participants in each included study varied from 68 to 27,349, age range 18–81 years with different cervical pathologies. The studies were of varying methodological quality, and were predominately performed in a secondary screening setting (i.e. women or cervical samples were subjected to a second test assay following a positive cytology or HPV-DNA. Among those participants who had cervical histological examination, the proportion of CIN2+ varied between 13.7 and 88.4%.

**Table 1:** Performance characteristics of the included studies 2012–2020

Authors	No. Part	Biomarkers	Current Status	Test platform	Sample type	Sensitivity	Specificity	PPV	NPV	AUC	Mean TAT
Farzanehpour et al, 2019 [2]	72	miRNA (miR-9)	Not validated	RT-qPCR	Tissue	64.7%	76.4%	–	–	0.71	≤ 5 h.
Farzanehpour et al. 2019 [2]	72	miRNA (miR-9)	Not validated	RT-qPCR	Serum	52.9%	94.4%	–	–	0.85	≤ 5 h.
Park et al., 2017 [11]	102	miRNA (miR-9)	Not validated	RT-qPCR	Tissue	67.3%	80%	77.7%	70.2%	0.76	≤ 5 h.
Wentzensen et al, 2012 [12]	673	p16INK4a / ki-67	Not validated	ICC CINtec PLUS	LBC	85.5%	59.4%	48.4%%	90.2%%	–	≤24 h
Zhu et al, 2019 [13]	300	p16INK4a / ki-67	Not validated	ICC CINtec PLUS	LBC	98.2%	82.5%	55.2%	99.5%%	0.90	≤24 h
Areán-Cun., et al, 2018 [14]	1945	p16INK4a / ki-67	Not validated	ICC CINtec PLUS	LBC	98%	39%	44.8%	97.5%	–	≤24 h
Wentzensen et al, 2015 [15]	1509	p16INK4a / ki-67	Not validated	ICC CINtec PLUS	LBC	83.4%	58.9%	21%	96.4%	–	≤24 h
Yu et al, 2016 [16]	231	p16INK4a / ki-67	Not validated	ICC CINtec PLUS	LBC	90.9%	79.5%	49.2%	97.6%	0.85	≤24 h
Tay et al., 2017 [17]	97	p16INK4a / ki-67	Not validated	ICC CINtec PLUS	LBC	92.9%	76.7%	88.1%	86.7%	0.85	≤24 h
Ebisch et al., 2017 [18]	462	p16INK4a / ki-67	Not validated	ICC CINtec PLUS	LBC	92%	61%	52%	95%	–	≤24 h
Hu et al., 2020 [19]	846	p16INK4a / ki-67	Not validated	ICC CINtec PLUS	LBC	86.5%	62.5%	28.8%	96.4%	–	≤24 h
Ordi et al., 2014 [20]	1169	p16INK4a / ki-67	Not validated	ICC CINtec PLUS	LBC	90.9%	72.1%	63.9%	93.6%	0.82	≤24 h
Uijterwaal et al., 2015 [21]	762	p16INK4a / ki-67	Not validated	ICC CINtec PLUS	LBC	68.8%	72.8%	25.2%	94.6%	–	≤24 h
White et al., 2016 [22]	1346	p16INK4a / ki-67	Not validated	ICC CINtec PLUS	LBC	75.4%	88.3%	26.6%	97%	–	≤24 h
Schmitz et al., 2018 [23]	280	p16INK4a / ki-67	Not validated	ICC CINtec PLUS	LBC	100%	90.2%	92.3%	100%	–	≤24 h
Han et al., 2020 [24]	468	p16INK4a / ki-67	Not validated	ICC CINtec PLUS	LBC	91.5%	77%	73.9%	92.8%	0.76	≤24 h
Li et al., 2020 [25]	4070	p16INK4a / ki-67	Not validated	ICC CINtec PLUS	LBC	90.9%	67%.	16.5%	99.1%	0.79	≤24 h
Wang et al., 2020 [26]	4070	p16INK4a / ki-67	Not validated	ICC CINtec PLUS	LBC	91.7%	63.5%	29.3%	97.9%	–	≤24 h
El-Zein et al., 2020 [27]	1649	p16INK4a / ki-67	Not validated	ICC CINtec PLUS	LBC	80.7%	69.4%	64%	71.8%	–	≤24 h
Wentzensen et al, 2019 [28]	3225	p16INK4a / ki-67	Not validated	ICC CINtec PLUS	LBC	82.8%	55.7%	24.3%	95%	–	≤24 h
Ren et al., 2019 [29]	300	p16INK4a / ki-67	Not validated	ICC CINtec PLUS	LBC	50%	75.3%	11.1%	96.1%	0.78	≤24 h
Bergeron, et al., 2015 [30]	27,349	p16INK4a / ki-67	Not validated	ICC CINtec PLUS	LBC	94.4%	78.7%	16.3%	99.7%	–	≤24 h
Polman et al., 2016 [31]	364	p16INK4a / ki-67	Not validated	ICC CINtec PLUS	LBC	69.2%	90.4%	51.9%	95.1%	–	≤24 h
Ren et al., 2019 [29]	300	HPV E6/E7 mRNA	Not validated	QuantiVirus ®HPV	LBC	100%	44.3%	10%	100%%	0.59	≤ 5 h.
Ren et al, 2018 [32]	160	HPV E6/E7 mRNA	Not validated	QuantiVirus®HPV	LBC	90.3%	49.6%	30.1%	95.5%	0.75	≤ 5 h
Han et al., 2018 [33]	6800	HPV E6/E7 mRNA	Not validated	QuantiVirus ®HPV	LBC	85.2%	66.7%	72.9%	81%	0.75	≤ 5 h
Yao et al., 2017 [34]	404	HPV E6/E7 mRNA	Not validated	QuantiVirus® HPV	LBC	89.5%	49%	39%	92.7%	0.72	≤ 5 h
Zhu et al, 2019 [13]	300	HPV E6/E7 mRNA	Not validated	QuantiVirus® HPV	LBC	87%	42.7%	25%	93.8%	0.70	≤ 5 h
Bountris et al, 2014 [35]	740	HPV E6/E7 mRNA	Not validated	NASBA assay	LBC	77%	90.2%	68.5%	93.4%	–	≤ 5 h
Li et al, 2016 [36]	186	HPV E6/E7 mRNA	Not validated	QuantiVirus® HPV	LBC	65%	86.7%	85.9%	66.7%	0.76	≤ 5 h
Camus et al, 2018 [37]	502	HPV E6/E7 mRNA	Not validated	qRT-PCR	LBC	90%	50%	64%	83%	0.80	≤ 5 h
Kong et al, 2020 [38]	600	DNA methylation	Not validated	qPCR	LBC	67.7%	94.9%	95.4%	65.5%	0.86	≤ 5 h
Dong et al, 2020 [39]	1997	DNA methylation	Not validated	qPCR	LBC	92.9%	73%	56.5%	96.4%	0.83	≤ 5 h
Schmitz et al, 2018 [23]	280	DNA methylation	Not validated	RT-PCR	LBC	59.7%	98%	91.5%	87.3%	–	≤ 5 h
van Leeuwen et al.,2019 [40]	262	DNA methylation	Not validated	qPCR	LBC	68%	67%	15%	96%	–	≤ 5 h
De Strooper et al, 2016 [41]	375	DNA methylation	Not validated	qPCR	LBC	70.5%	67.8%	35.5%	90.2%	–	≤ 5 h
Chujan et al, 2014 [42]	94	DNA methylation	Not validated	qPCR	LBC	83.3%	96.8%	92.6%	92.5%	–	≤ 5 h
Kottaridi et al, 2017 [43]	151	DNA methylation	Not validated	qPCR	LBC	75.7%	77.5%	74.7%	78.5%	0.81	≤ 5 h
Leeman et al, 2019 [44]	262	DNA methylation	Not validated	qPCR	LCB	77.8%	69.3%	36.4%	98.3%	–	≤ 5 h
Chen et al, 2020 [45]	103	SCC-Ag	Validated	Elisa	Serum	80%	100%	100%	82.6%	0.89	≤ 5ihrs
Zajkowska et al, 2018 [46]	100	SCC-Ag	Validated	Elisa	Serum	78.8%	74%	66.7%	84.1%	0.79	≤ 5 h
Sidorkiewicz et al., 2019 [5]	85	SCC-Ag	Validated	Elisa	Serum	81.2%	74%	–	–	0.79	≤ 5 h
Lubowicka et al,2020 [47]	89	SCC-Ag	Validated	Elisa	Serum	78.6%	74%	–	–	0.79	≤ 5 h
Zajkowska et al, 2018 [46]	85	M-CSF	Not validated	Elisa	Serum	72.7%	86%	77.4%	82.7%	0.79	≤ 5 h
Lubowicka et al, 2020 [47]	89	M-CSF	Not validated	Elisa	Serum	75%	86%	–	–	0.81	≤ 5 h
Sidorkiewicz et al., 2019 [5]	85	M-CSF	Not validated	Elisa	serum	69.4%	86%	–	–	0.81	≤ 5 h
Ruan et al, 2020 [48]	68	M-CSF	Not validated	Elisa	Serum	87.7%	64.7%	–	–	0.75	≤ 5 h
Będkowska et al., 2015 [49]	110	M-CSF	Not validated	Elisa	Serum	68%	94%	92%	75%	0.86	≤ 5 h
Lawicki et al, 2016 [50]	100	VEGF	Not validated	Elisa	Serum	56%	96%	86%	82%	0.85	≤ 5 h
Sidorkiewicz et al.,2019 [5]	85	VEGF	Not validated	Elisa	Serum	81.2%	76%	–	–	0.86	≤ 5 h
Cheng et al., 2013 [51]	109	VEGF	Not validated	Elisa	Serum	83.3%	74.6%	–	–	0.83	≤ 5 h
Urquidi et al, 2012 [52]	127	VEGF	Not validated	Elisa	Urine	83%	87%	–	–	0.88	≤ 5 h

*DNA* Deoxyribonucleic Acid, *HPV* E6/7 Human Papilloma Virus Early protein 6/7, *mRNA* Messenger ribonucleic acid, *NPV* Negative Predictive Value, *RT PCR* Reverse Transcriptase Polymerase Chain Reaction, *PPV* Positive Predictive Value, *AUC* Area Under Receiver Operating Characteristic Curve, *SCC-Ag* Squamous Cell Carcinoma Antigen, *M-CSF* Macrophage Colony Stimulating Factor, *VEGF* Vascular Endothelial Growth Factor, *LBC* Liquid Based Cytology, *TAT* Turnaround Time, *miRNA-9* micro ribonucleic acid-9, *Elisa* Enzyme Linked Immunosorbent Assay. No Part: Number of Participants; *ICC* Immunocytochemistry; *NASBA* Nucleic acid sequence based amplification for the identification of E6/E7 mRNA of the HPV types; (−) means not done; Validated: means approved for use; Not validated means investigation in progress

### Risk of bias

Ratings of the study quality for each of the nine domain was based on critical appraisal skills program (CASP) [10] criteria presented in Additional file 2. The risk of bias for each individual domain was rated as ‘Yes’, ‘No’ or ‘Can’t tell’. The assessment of quality results was categorized not scored otherwise. Overall, we included studies which had no major methodological anomalies.

### Diagnostic performance of the listed assays to detect CIN2+

Owing to the difference in clinical presentation of subjects, pooling the diagnostic performance data was challenging. In its place, we compiled best evidence synthesis for each of the assays to detect high-grade cervical lesions (CIN2+) using descriptive statistics. The diagnostic performance of each of the assays as compared with histologically confirmed high-grade cervical intraepithelial neoplasia (CIN2+) as an endpoint was as follows; 1) HPV E6/E7 mRNA test performance varied considerably depending on the diagnostic method used as shown in Table 1. Eight of the studies included recorded a sensitivity, specificity, PPV, NPV, and Area Under Receiver Operating Characteristic Curve (AUC) ranges of 65–100%, 42.7–90.2%, 10–85.9%, 66.7–100%, and 0.59–0.80, respectively, using liquid based cytology (LBC) samples majorly on QuantiVirus®HPV platform [13, 29, 32–37]. 2). For miRNA (miR-9) assay, three studies recorded sensitivity, specificity and area under the ROC curve ranges of 52.9–67.3%, 76.4–94.4%%, and 0.71–0.85, respectively based on RT-qPCR platform using serum and tissue samples [2, 11, 53], with77.8% PPV and 70.2% NPV reported by *Park* et al [11]. 3) For the p16INK4a / ki-67 assays, twenty studies included reported sensitivity, specificity, PPV, NPV, and AUC ranges of 50–100%, 39–90.4%, 11.1–92.3%, 86.7–100%, and 0.76–0.90, respectively, based on immunocytochemistry (ICC) platform using liquid based cytology (LBC) samples [12–30]. 4) Eight studies evaluating DNA methylation assays reported sensitivity, specificity, PPV, NPV and AUC ranges of 59.7–92.9%, 67–98%, 15–95.4%, 65.5–98.3%, and 0.81–0.86, respectively, in detection of CIN2+ based on RT-PCR platform using LBC samples [23, 38–44].5). Meanwhile, four studies included in our review evaluating Squamous Cell carcinoma Antigen (SCC-Ag) assays recorded sensitivity, specificity, PPV, NPV and AUC ranges of 78.6–81.2%, 74–100%, 66.7–100%, 82.6–84.1%, and 0.79–0.89, respectively, in detection of CIN2+ based on Elisa platform using serum samples [5, 45–47]. 6). Five studies evaluating Macrophage Colony Stimulating Factor (M-CSF) assays included in our review reported sensitivity, specificity, PPV, NPV, and AUC ranges of 68–87.7%, 64.7–94%,77.4–92%, 75–82.7%, and 0.79–0.86, respectively, based on Elisa platform using serum; with Zajkowska et al*, and* Bedkowska et al being the only authors who reported PPVs and NPVs at 77.4, 92 and 82.7%, 75%, respectively, [5, 46–49].7). On the other hand, four studies evaluating Vascular Endothelial Growth Factor (VEGF) assay reported sensitivity, specificity, and AUC ranges of 56–83.5%, 74.6–96%, and 0.83–0.88, respectively, based on Elisa platform using serum [5, 50–52], with Lawicki et al being the only author who reported PPV and NPV at 86 and 82%, respectively.

Overall, Table 1 is a summary of the performance characteristics for each of the 58 studies included. The area under the ROC curve and NPV indicates the clinical usefulness of a tumor marker. In this review, the area under the ROC curve of p16INK4a / ki-67 assay was the largest, with highest NPV among the assays evaluated. Other assays recorded relatively similar area under the receiver operating characteristic (ROC) curve for diagnosis of high-grade cervical lesions (CIN2+) considering histology as gold standard. The mean turnaround time for all serum and LBC assays was ≤5 h, except for immunocytochemistry that recorded a mean TAT of 24 h.

## Discussion

Similar reviews on the performance characteristics of some of the listed assays had been reported earlier by Tornesello et al*,* Shah et al.*, and* American Society for Clinical Pathology [6–8]*.* Our review is therefore an update of the latest knowledge on the test performance of these assays compiled from articles published since 2012, some of which included a number of studies with varying methodological quality, but our finding is in line with this review. Accordingly, together with the previous reviews [6–8], our finding would be considered for further large scale studies to generate bold data on the clinical applicability of some of these assays. In the present review, women were tested for the HPV E6/E7 mRNA, miRNA (miR-9), p16INK4a / ki-67, DNA methylation, SCC-Ag, M-CSF, and VEGF predominately secondary to having positive cervical cytology, and/or VIA and/or positive HPV DNA test. Overexpression of E6 / E7, p16 /ki-67, miR-9, SCC-Ag, M-CSF, VEGF proteins, or JAM3, SOX1, and L1 genes following infection with HPV can be detected based on their elevated levels in plasma, serum, Cervical scraping, or tissue as predictors of increased risk of cervical cancer progression [29, 32, 54, 55]. The proportion of CIN2+ varied between 13.7 and 88.4%, reflecting the diverse spectrum of cervical pathologies of the participants employed in articles we included.

From experimental studies, it has been established that woman exposed to HPV E6/E7 mRNA following infection with HPV have higher risk of progressing to high-grade cervical neoplasia due to the integration of viral DNA sequence into host genome causing loss of E2 tumor suppressor gene that regulates expression of E6 and E7 oncogenes [13, 54, 56]. Consequently, this results in overexpression of the two oncogenes which become useful in evaluating risk of cervical carcinogenesis. This consensus is supported by studies included in our review showing that HPV E6/E7 mRNA assays have diagnostic relevance for CIN2+ with sensitivity, specificity, PPV, NPV, and AUC ranges of 65–100%, 42.7–90.2%, 10–85.9%, 66.7–100%, and 0.59–0.80, respectively, [13, 29, 32, 33]. However, due to the heterogeneity of participants in the included studies, the results of HPV E6/E7 mRNA test performance have limited generalizability. Moreover, a number of studies also produced varying diagnostic results with extreme specificity of 42,7% reported by Zhu et al*,* [13], compared to 90.2% reported by Bountris et al, [35]; and extreme PPV of 10% reported by Ren et al [29], compared to 85.9% PPV by Li et al [36]. Similarly, a smaller area under receiver operating characteristic curve (AUC) of 0.59 was reported by Ren et al [29], compared to 0.80 reported by Camus et al. [37], of which the disparity might have resulted from a difference in the type of included study participants who had different cervical pathologies. Although our findings are in agreement with a similar review by Macedo et al, which recorded pooled sensitivity, specificity and AUC of 92.8, 60.5% and 0.88, respectively, [56], more robust clinical translation studies using larger consecutive cohorts of women participants is recommended for adequate validation.

Meanwhile, our review of the performance characteristic of microRNAs (miRNA) assays, particularly miR-9 in detection of CIN2+ recorded sensitivity, specificity and area under the ROC curve ranges of 52.9–67.3%%, 76.4–94.4%, and 0.71–0.85, respectively, [2, 11] with 77.7% PPV and 70.2% NPV reported by Park et al [11]. The high specificity ranges recorded from our review proved that miR-9 assay have diagnostic relevance to detect CIN2+. Although predictive values were missing in a number of studies included, sensitivity and specificity appeared to be similar across, and consistent with earlier review conducted by Jiang et al which recorded sensitivity, specificity, and AUC of 73, 94%, and 0.95, respectively, with 13.2 Positive Likelihood Ratio (PLR), and 0.28 Negative Likelihood Ratio (NLR) [53]. Experimental studies have shown that epigenetic instability is greatly influenced by miRNA which plays important role in transcriptional regulation, and any form of dysregulation as seen in overexpression often lead to a wide range of human malignancy including cervical cancer [2, 3, 11]. Like many other circulating miRNAs, studies have established that miR-9 could be useful for early detection of cervical cancer, predicting cancer prognosis, and in monitoring clinical outcome of cancer disease [55]. And that, by examining the associations between miR-9 levels in exfoliated cells, cervical tissues or serum; and the diverse biological processes such as metabolism and apoptosis, there is a consensus across studies showing that elevated levels is valuable for evaluating risk of cervical Intraepithelial neoplasia (CIN) in suspected individuals [2, 7, 53, 55, 57, 58], especially in conjunction with other equally useful markers such as miR-21, miR-155, miR-192, miR-203 and miR-205 to improve specificity for optimal treatment benefit [2, 11, 48, 53, 59–61]. Although our review is in agreement with this general consensus on diagnostic relevance of miR-9 in detection of CIN2+ [2, 11], coupled with reduced turnaround time (TAT), and non-invasive blood sampling [53, 55]; more robust clinical translation studies with larger consecutive cohorts of women participants would be appropriate for adequate validation alongside cost evaluation prior to implementation.

Moreover, in our review of p16INK4a / ki-67 assays, twenty studies recorded sensitivity, specificity, PPV, NPV, and AUC ranges of 50–100%, 39–90.4%, 11.1–92.3%, 86.7–100%, and 0.76–0.90, respectively, for the detection of CIN2+ [12, 13, 23, 28–30], with lower sensitivity of 50% reported by Ren et al [29], compared to100% sensitivity by Schmitz et al [23]. Equally, extreme specificity of 39% was reported by Areán-Cun et al. [14], compared to 90.4% specificity by Polman et al [31]*, and* extreme PPV of 11.1% reported by Ren et al. [29] compared to 92.3% PPV by Schmitz et al. [23], with area under the ROC curve similar across studies included [13, 16, 17, 29]. Although considerable variations in diagnostic performance was observed among different studies owing to differences in population background such as age, race and methods of cytology testing, the ability of p16INK4a / ki-67 assay to detect correctly women without cervical neoplasia was applaudable; especially with larger Area Under (ROC) Curve, and higher NPV observed across studies included, coupled with convenient self sampling, non-intrusiveness, and reduced turnaround time (TAT) Table 1. Our findings are consistent with earlier review by Sun et al which recorded similar sensitivity and specificity ranges of 68.8–94.4 and 30.6–95.2, respectively, [62]. Studies have shown that detection of Ki-67 (MIB-1) nuclear biomarker and p16ink4a cytoplasmic biomarker in cervical epithelial cells is useful in detection of Low-grade squamous intraepithelial lesion (LSIL) [31], and can help to predict the prognosis of which cases of Atypical Squamous Cells of Undetermined Significance (ASCUS) and LSIL will progress to High-grade squamous intraepithelial lesion (HSIL), and invasive cancer. Hence, integration of p16ink4a / ki-67 assay as a point of care test to be used specifically to identify at one visit, cases of cervical dysplasia with subsequent cryotherapy treatment, is an option extensively under investigation [28, 31, 63]. Recent studies have recommended the implementation of p16/Ki-67 and HPV-DNA tests combination for safe monitoring the recurrence of CIN2+ given that some patients treated for CIN2 and CIN3 tend to relapse overtime [28]. However, there is a serious doubt as to whether a combination p16/Ki-67 assay and VIA may serve as alternative in facilities with resource limitations considering low sensitivity associated with VIA [63, 64].

Accordingly, this review established that p16/Ki-67 assay is preferable for triaging HPV-DNA or VIA positives cases given the robust clinical translation studies with larger consecutive cohorts of women participants recorded in the recent past [30]. Moreover, studies have also demonstrated that p16/Ki-67 assay is able to identify accurately women at risk of precancerous lesions who may need to undergo further retesting at extended intervals [31]. Thus, with minimal training on the staining and interpretation protocol as demonstrated earlier in Slovenia and California [65–67], other cytotechnologists and cytopathologists in low and middle income countries (LMIC) would equally be able to examine and report correctly cases of cervical neoplasia given that similar trainings had also been piloted in Kenya and Malawi with good results [68]. Furthermore, considering the interobservers variability, and the need for repeat tests associated with Pap cytology, studies have equally shown that implementation of p16/Ki-67 assay would be more cost effective compared to the conventional Pap cytology [69].

DNA methylation assays on the other hand recorded sensitivity, specificity, PPV, NPV, and AUC ranges of 59.7–92.9%, 67–98%, 15–95.4%, 65.5–98.3%, and 0.81–0.86, respectively, in detection of CIN2+ [23, 38–44], with a low sensitivity of 59.7% reported by Schmitz et al [23], compared to 92.9% sensitivity by *Dong* et al [39]*;* and extreme PPV of 15% reported by van Leeuwen et al [40], compared to 95.4% PPV by Kong et al, [38]. DNA methylation is a major epigenetic mechanism that involves the transfer of a methyl group to the C5 carbon residues (5mC) of cytosines that is mediated by a family of DNA methyltransferases, and plays an important role in various biological processes including the regulation of gene expression, genomic imprinting, cell differentiation, development, and inflammation [70]. Studies have shown that DNA hypermethylation may occur when multiple methyl groups are transferred to one cytosine that should not be methylated, causing gene silencing with subsequent initiation of carcinogenesis [13]. Given that DNA methylation is significantly higher in CIN2+ and CIN3+ women, determining levels of key genes such as JAM3, SOX1 or L1 in cytology samples as a triage test for HPV positive women is recommended owing to higher specificity compared to cytology Atypical Squamous Cell of Undetermined Significance (ASCUS), and sensitivity higher than HPV16/18 genotyping [39]. From our review findings, the higher specificity and area under the ROC curve recorded from various studies, coupled with convenient LBC sampling and shorter TAT support the DNA methylation suitability for facilities with no established histology infrastructure, subject to more robust clinical translation studies with larger consecutive cohorts of women participants.

Four studies evaluating Squamous Cell carcinoma Antigen (SCC-Ag) assays recorded sensitivity, specificity, PPV, NPV and AUC ranges of 78.6–81.2%, 74–100%, 66.7–100%, 82.6–84.1%, and 0.79–0.89, respectively, in detection of CIN2+ [5, 45–47], with similar performance observed across studies included. Although SCC-Ag assay is currently implemented as a rapid screening test for women at high risk of cervical neoplasia [4], and as a prognostic tool for monitoring recurrent uterine cervical cancer following a concurrent Chemoradiotherapy (CCRT) [45, 71], studies have recommended the measurements of SCC-Ag serum levels in conjunction with other complementary markers such M-CSF or VEGF to improve specificity for optimal treatment benefit [46]. Squamous cell carcinoma (SCC) antigen belongs to the serine protease inhibitor (Serpin) family of proteins that have been confirmed as tumor markers for cervical squamous cell carcinoma, and is often seen elevated in patients serum suggestive of tumor stage, parametrial invasion, and lymph node metastasis [45, 72]. It is present at high levels in 20–60% of patients with early stage cervical cancer (CC), with abnormally high levels also observed in 25% of individuals with adenocarcinoma (ADC) [7]. Recent studies have established that elevated levels of SCC-Ag is associated with extensive tumor, poor survival of patients treated by CCRT, and radiotherapy resistance [71, 73]; and that, preoperative SCC-Ag is equally useful in predicting adjuvant chemotherapy outcome in patients with intermediate-risk factors [74]. Unfortunately, few studies have evaluated the performance of SCC-Ag assay, either as single biomarker or in combination with other complementary biomarkers; thus, calling for more robust clinical translation studies with larger consecutive cohorts of women participants.

Macrophage Colony Stimulating Factor (M-CSF) assays included in our review reported sensitivity, specificity, PPV, NPV, and AUC ranges of 68–87.7%, 64.7–94%,77.4–92%, 75–82.7%, and 0.75–0.86, respectively, [5, 46–49], with performance characteristics relatively similar across all studies. Macrophage colony-stimulating factor (M-CSF) is a hematopoietic growth factor that stimulates the proliferation and differentiation of Monocytes to macrophages. Experimental studies have shown that increased expression of M-CSF and its receptor leads to recruitment of tumor-associated macrophages (TAMs) in different types of cancers that also stimulate cancer cell proliferation and migration [5, 47, 48]. Consequently, M-CSF overexpression in plasma levels serves as useful predictor of carcinogenesis, and poor prognosis [46]. Although M-CSF assay displays useful diagnostic values for CIN2+, Lubowicka et al.*, and* Zajkowska et al recommended the interpretation of elevated levels in conjunction with other complementary markers such as VEGF or SCC-Ag. to improve specificity for optimal treatment benefit, given that their levels are equally raised in other types of cancer as well [47]; a suggestion also supported by Sidorkiewicz et al [5]. Although our review results are in agreement with included studies, more robust clinical translation studies with larger consecutive cohorts of women participants would be appropriate for adequate validation of the assay.

Finally, four of the studies evaluating Vascular Endothelial Growth Factor (VEGF) assays included in our review recorded sensitivity, specificity, and AUC ranges of 56–83.5%, 74.6–96%, and 0.83–0.86, respectively, [5, 50–52], with 86% PPV and 82% NPV reported by Ławicki, et al. [50]. Our review findings supports earlier suggestion by Sidorkiewicz et al.and Cheng et al*,* of the diagnostic usefulness, and clinical applicability of VEGF assay in cervical, breast or endometrial cancer, particularly with regards to consistency in specificity and AUC across all studies; and the diagnostic correlation with other complementary assays such as M-CSF and SCC-Ag [5, 51]. Members of Vascular Endothelial Growth Factor (VEGF) family comprising VEGF-A, −B, −C, −D, and placenta growth factor (PlGF) are dimeric glycoprotein measuring 34–42 kDa, and constitutes one of the most important signaling pathways associated with angiogenesis [75]. Although VEGF biomolecules normally express in normal cells, elevated levels in plasma has been associated with cervical or endometrial cancer [5, 51, 75]. Ceci et al, in his review observed that patient with VEGF overexpression often present with bulky tumors, pelvic lymph node involvement and parametrial infiltration [75], an observation equally supported by Zusterzeel et al. [76]. Accordingly, our review results supported earlier suggestion of clinical usefulness of VEGF in the diagnosis of cervical cancer; subject to more robust clinical translation studies with larger consecutive cohorts of women participants.

### Limitations

This systematic review presents the latest developments in the field of SCC Ag, M-CSF, VEGF, miRNA (miR-9), p16INKa / ki-67, HPV E6/E7 mRNA and DNA methylation tests accuracy. We have included relatively adequate number of articles published in different countries employing large number of study participants. However, our review result should be interpreted in light of a few shortcomings. Our main setback was lack of studies that employed similar and well-defined population with same cervical pathology characteristics. Thus, our review suffered from heterogeneity of studies which made it difficult to pool the performance characteristics of each of the tested assays. Additionally, use of histologically confirmed CIN2+ endpoint when evaluating the test accuracy represents a challenge because of the regression (false positive) or progression (false negative) of many confirmed lesions. Moreover, confining our inclusion criteria to include only articles published in English languages would also mean missing some of the relevant studies; thus reducing the accuracy of our results.

## Conclusions

The larger AUC and higher NPV correspond to a better diagnostic tool. Consequently, the reported test performance and the receiving operating characteristics curves implies that implementation of p16ink4a / ki-67 assay as a point of care test to be used specifically to triage HPV-DNA positive women at one visit with subsequent cryotherapy treatment is feasible, especially in regions with inadequate histology infrastructure such as Kenya [68, 77]. This will reduce colposcopy referrals [78], and cushion high loss to follow-up associated with histology longer turnaround time [78, 79]. For the rest of assays, more robust clinical translation studies with larger consecutive cohorts of women participants is recommended for adequate validation, coupled with cost evaluation prior to implementation.

## Supplementary Information

**Additional file 1.**

**Additional file 2.**

## Data Availability

All the generated data in this review are included in the manuscript.

## Abbreviations

DNA: Deoxyribonucleic Acid
HPV E6/7: Human Papilloma Virus Early protein 6/7
mRNA: Messenger ribonucleic acid
NPV: Negative Predictive
RT- PCR: Reverse Transcriptase Polymerase Chain Reaction
PPV: Positive Predictive Value
AUC: Area Under Receiver Operating Characteristic Curve
SCC Ag: Squamous Cell Carcinoma Antigen
M-CSF: Macrophage Colony Stimulating Factor
VEGF: Vascular Endothelial Growth Factor
LBC: Liquid Based Cytology
TAT: Turnaround Time
miRNA-9: Micro ribonucleic acid-9
Elisa: Enzyme Linked Immunosorbent Assay
CIN2+: Cervical intraepithelial Neoplasia 2 and above
VIA: Visual Inspection with acetic Acid
HPV: Human Papilloma Virus
ICC: Immunocytochemistry
LSIL: Low-grade squamous intraepithelial lesion
HSIL: High-grade squamous intraepithelial lesion

## References

[1] WHO (2018). Global Cancer Statistics 2018: GLOBOCAN Estimates of Incidence and Mortality Worldwide for 36 Cancers in 185 Countries. CA Cancer J Clin.

[2] Farzanehpour M, Mozhgani SH, Somayeh Jalilvand S, Faghihloo E, Setareh Akhavan S, Vahid Salimi V, Talat Mokhtari Azad TM (2019). Serum and tissue miRNAs: potential biomarkers for the diagnosis of cervical cancer. J Virol.

[3] Ma X, Lakshmipriya T, Gopinath SCB (2019). Recent Advances in Identifying Biomarkers and High-Affinity Aptamers for Gynecologic Cancers Diagnosis and Therapy. Hindawi J Anal Methods Chem.

[4] Dasari S, Wudayagiri R, Valluru L (2015). Cervical cancer: biomarkers for diagnosis and treatment. Clin Chim Acta.

[5] Sidorkiewicz I, Zbucka-Krętowska M, Zaręba K, Lubowicka E, Zajkowska M, Szmitkowski M, Gacuta E, Ławicki S (2019). Plasma levels of M-CSF and VEGF in laboratory diagnostics and differentiation of selected histological types of cervical cancers. BMC Cancer.

[6] Tornesello ML, Buonaguro L, Giorgi-Rossi P, Buonaguro FM (2013). Viral and Cellular Biomarkers in the Diagnosis of Cervical Intraepithelial Neoplasia and Cancer. BioMed Res Int.

[7] ASCP, A.S.f.C.P, Laengsri V, Kerdpin U, Plabplueng C, Treeratanapiboon L, Nuchnoi P. Cervical Cancer Markers: Epigenetics and microRNAs. Lab Med. 2018;49:97–111. Available from: 10.1093/labmed/lmx080. www.labmedicine.com. Accessed 30 Sept 2020. 10.1093/labmed/lmx080 29378033

[8] Shah UJ, Nasiruddinb M, Dard SA, Khane KA, Akhterc MR (2020). Emerging biomarkers and clinical significance of HPV genotyping in prevention and management of cervical cancer. Microb Pathog.

[9] Shamseer L, Mother D, Clarke M, Ghersi D, Liberati A, Petticrew M, Shekelle P, Stewart LA (2015). Preferred reporting items for systematic review and meta-analysis protocols (PRISMA-P) 2015: elaboration and explanation. BMJ.

[10] CASP, Critical Appraisal Skills Programme. 2018 ad/2a-casp_cohort_tool.pdf. Available from: https://www.unisa.edu.au/contentassets/72bf75606a2b4abcaf7f17404af374ad/2a-casp_cohort_tool.pdf. Accessed 30 Sept 2020.

[11] Park S, Eom K, Kim J, Bang H, Wang H, Ahn S, Kim G, Jang H, Kim S, Lee D, Park KH, Lee H (2017). MiR-9, miR-21, and miR-155 as potential biomarkers for HPV positive and negative cervical cancer. BMC Cancer.

[12] Wentzensen N, Schwartz L, Zuna RE, Smith K, Mathews C, Gold MA, Allen RA, Zhang R, Dunn ST, Walker JL, Schiffman M (2012). Performance of p16/Ki-67 Immunostaining to detect Cervica Cancer precursors in a colposcopy referral population. Clin Cancer Res.

[13] Zhu Y, Ren C, Yang L, Zhang X, Liu L, Wang Z (2019). Performance of p16/Ki67 immunostaining HPV E6/E7 mRNA testing, and HPV DNA assay to detect high-grade cervical dysplasia in women with ASCUS. BMC Cancer.

[14] Areán-Cuns C, Mercado-Gutiérrez M, Paniello-Alastruey I, Mallor-Giménez F, Córdoba-Iturriagagoitia A, Lozano-Escario M, Santamaria-Martínez M. Dual staining for p16/Ki67 is a more specific test than cytology for triage of HPV-positive women. Virchows Archiv. 2018;473:599–606. Available from: 10.1007/s00428-018-2432-z. Accessed 30 Sept 2020. 10.1007/s00428-018-2432-z 30094492

[15] Wentzensen N, Fetterman B, Castle PE, Schiffman M, Wood SN, Stiemerling E, Tokugawa D, Bodelon C, Poitras N, Lorey T, Kinney W (2015). p16/Ki-67 Dual Stain Cytology for Detection of Cervical Precancer in HPV-Positive Women. J Natl Cancer Inst.

[16] Yu LL, Chen W, Lei XQ, Qin Y, Wu ZN, Pan QJ. Evaluation of p16/Ki-67 dual staining in detection of cervical precancer and cancers: a multicenter study in China. Oncotarget. 2016;7(16):21181–9. Available from: www.impactjournals.com/oncotarget/. Accessed 30 Sept 2020. 10.18632/oncotarget.8307 PMC5008277 27029033

[17] Tay TKY, Lim KL, Hilmy MH, Thike AA, Thoe S, Song LH, Hwang JSG, Manto S (2017). Comparison of the sensitivity and specificity of p16/Ki-67 dual staining and HPV DNA testing of abnormal cervical cytology in the detection of histology proven cervical intraepithelial neoplasia grade 2 and above (CIN 2+). Malays J Pathol.

[18] Ebisch RMF, van der Horst J, Hermsen M, Rijstenberg L, Vedder JEM, Bulten J (2017). Evaluation of p16/Ki-67 dual-stained cytology as triage test for high-risk human papillomavirus-positive women. Mod Pathol.

[19] Hu Y, Hong Z, Gu L, Xie L, Yang B, Dai H, Chen H, Zhang B (2020). Evaluation of p16/Ki-67 dual-stained cytology in triaging HPV-positive women during cervical Cancer screening. Cancer Epidemiol Biomarkers Prev.

[20] Ordi J, Sagasta A, Munmany M, Rodríguez-Carunchio LR, Torn A, del Pino M. Usefulness of p16/Ki67 Immunostaining in the Triage of Women Referred to Colposcopy. Cancer Cytopathol. 2014;122(3):227–35. Available from: 10.1002/cncy.21366wileyonlinelibrary.com. Accessed 30 Sept 2020. 10.1002/cncy.21366 24757722

[21] Uijterwaal MH, Polman NJ, Witte BI, van Kemenade FJ, Rijkaart D, Berkhof J (2015). Triaging HPV-positive women with normal cytology by p16/Ki-67 dual-stained cytology testing: Baseline and longitudinal data. Int J Cancer.

[22] White C, Bakhiet S, Bates M, Keegan H, Pilkington L, Ruttle C, Sharp L (2016). Triage of LSIL/ASC-US with p16/Ki-67 dual staining and human papillomavirus testing: a 2-year prospective study. Cytopathology.

[23] Schmitz M, Eichelkraut K, Schmidt D, Zeiser I, Hilal Z, Tettenborn Z, Hansel A, Ikenberg H (2018). Performance of a DNA methylation marker panel using liquid-based cervical scrapes to detect cervical cancer and its precancerous stages. BMC Cancer.

[24] Han Q, Guo H, Geng L, Wang Y (2020). p16/Ki-67 dual-stained cytology used for triage in cervical cancer opportunistic screening. Chin J Cancer Res.

[25] Li YC, Zhao YQ, Li TY, Chen W, Liao GD, Wang HR, Lei HK, Guo Y, Zhou Q (2020). The Performance of Immunocytochemistry Staining as Triaging Tests for High-Risk HPV-Positive Women: A 24-Month Prospective Study. J Oncol.

[26] Wang HR, Li YC, Guo HQ, Yu LL, Wu Z, Yin J, Liao GD, Qu YM, Jiang Y, Wang D, Chen W. A cocktail of p16INK4a and Ki-67, p16INK4a and minichromosome maintenance protein 2 as triage tests for human papillomavirus primary cervical cancer screening. Oncotarget 2017;8(48):83890–99. Available from: www.impactjournals.com/oncotarget/. Accessed 15 Oct 2020. 10.18632/oncotarget.19870 PMC5663562 29137390

[27] El-Zein M, Gotlieb W, Gilbert L, Hemmings R, Behr MA, Franco EL. Tumor Markers and Signatures Dual staining for p16/Ki-67 to detect high-grade cervicallesions: Results from the Screening Triage Ascertaining Intraepithelial Neoplasia by Immunostain Testing study. Int J Cancer. 2020:1–10. Available from: 10.1002/ijc.33250. Accessed 30 Sept 2020. 10.1002/ijc.33250 32781481

[28] Wentzensen N, Clarke MA, Bremer R, Poitras N, Tokugawa D, Goldhoff PE, Castle PE, Schiffman M, Kingery JD, Grewal KK, Locke A, Kinney W, Lorey TS (2019). Clinical Evaluation of Human Papillomavirus Screening With p16/Ki-67 Dual Stain Triage in a Large Organized Cervical Cancer Screening Program. JAMA Intern Med.

[29] Ren C, Zhu Y, Yang L, Zhang X, Liu L, Wang Z, Jiang D (2019). Prognostic and diagnostic validity of p16/ Ki-67, HPV E6/E7 mRNA, and HPV DNA in women with ASCUS: a follow-up study. Virol J.

[30] Bergeron C, Ikenberg H, Sideri M, Denton K, Bogers J, Schmidt D, Francisco Alameda F. Prospective Evaluation of p16/Ki-67 Dual-Stained Cytology for Managing Women With Abnormal Papanicolaou Cytology: PALMS Study Results. Cancer Cytopathol. 2015;123(6):373–81. Available from: 10.1002/cncy.21542. www.wileyonlinelibrary.com. Accessed 30 Sept 2020. 10.1002/cncy.21542 25891096

[31] Polman NJ, Uijterwaal MH, Witte B, Berkhof J, van Kemenade FJ, Spruijt JWM (2017). Good performance of p16/ki-67 dual-stained cytology for surveillance of women treated for high-grade CIN. Int J Cancer.

[32] Ren C, Zhu Y, Yang L, Zhang X, Liu L, Ren C (2018). Diagnostic performance of HPV E6/E7 mRNA assay for detection of cervical high-grade intraepithelial neoplasia and cancer among women with ASCUS Papanicolaou smears. Gynecol Obstet.

[33] Han L, Husaiyin S, Zhao F, Rezhake R, Niyazi M. Clinical Value of Human Papillomavirus E6/E7 mRNA Detection in Screening for Cervical Cancer in Women Positive for Human Papillomavirus DNA. Clin Lab. 2018;64(9):1363–71. Available from: 10.7754/Clin.Lab.2018.180138. Accessed 30 Sept 2020. 10.7754/Clin.Lab.2018.180138 30274003

[34] Yao Y, Ian Q, Cheng B, Cheng Y, Ye J, Lu W (2017). Human papillomavirus (HPV) E6/E7 mRNA detection in cervical exfoliated cells: a potential triage for HPV-positive women*. J Zhejiang Univ Sci B.

[35] Bountris P, Haritou M, Pouliakis A, Margari N, Kyrgiou M. An Intelligent Clinical Decision Support System for Patient-Specific Predictions to Improve Cervical Intraepithelial Neoplasia Detection. BioMed Res Int. 2014;341483:20. Available from: 10.1155/2014/341483. Accessed 30 Sept 2020. . 10.1155/2014/341483 PMC4000928 24812614

[36] Li J, Yi JL, Li Q, Zhang W, Li Y, Qu PP, Wei YL (2016). Risk evaluation of cervical cancer progress by screening human papillomavirus DNA, E6/E7 mRNA and protein, and cell free ferrous protoporphyrin. Int J Clin Exp Med.

[37] Camus C, Vitale S, Loubatier C, Pénaranda G, Khiri H (2018). Quantification of HPV16 E6/E7 mRNA Spliced Isoforms Viral Load as a Novel Diagnostic Tool for Improving Cervical Cancer Screening. J Clin Med.

[38] Kong L, Wang L, Wang Z, Xia X, You Y, Wu H, Wu M, Liu P, Li L (2020). DNA methylation for cervical cancer screening: a training set in China. Clin Epigenetics.

[39] Dong L, Zhang L, Hu SY, Feng RM, Zha XL, Zhang Q, Pan QJ, Zhang X, You-Lin Qiao YL, Fang-Hui Zhao FH (2020). Risk stratification of HPV 16 DNA methylation combined with E6 oncoprotein in cervical cancer screening: a 10-year prospective cohort study. Clin Epigenetics.

[40] van Leeuwen RW, Oštrbenk A, Poljak M, van der Zee AG, Schuuring E, Wisman GBA (2019). DNA methylation markers as a triage test for identification of cervical lesions in a high risk human papillomavirus positive screening cohort. Int J Cancer.

[41] De Strooper LMA, Verhoef VMJ, Berkhof J, Hesselink AT, de Bruin HME (2016). Validation of the FAM19A4/mir124–2 DNA methylation test for both lavage- and brush-based self-samples to detect cervical (pre) cancer in HPV-positive women. Gynecol Oncol.

[42] Chujan S, Kitkumthorn N, Siriangku S, Mutirangura A (2014). CCNA1 promoter methylation: a potential marker for grading Papanicolaou smear cervical squamous intraepithelial lesions. Asian Pac J Cancer Prev.

[43] Kottaridi C, Kyrgiou M, Pouliakis A, Magkana M, Aga E, Spathis A, Mitra A (2017). Quantitative measurement of L1 human papillomavirus type 16 methylation for the prediction of Preinvasive and invasive cervical disease. J Infect Dis.

[44] Leeman A, del Pino M, Marimon L, Torné A, Ordi J, ter Harmsel B (2019). Reliable identification of women with CIN3+ using hrHPV genotyping and methylation markers in a cytology-screened referral population. Int J Cancer.

[45] Chen P, Jiao L, Ren F, Wang DB. Clinical value of serum squamous cell carcinoma antigen levels in predicting chemosensitivity, lymph node metastasis, and prognosis in patients with cervical squamous cell carcinoma. BMC Cancer. 2020;20:423. 10.1186/s12885-020-06934-x. 10.1186/s12885-020-06934-x PMC7227060 32410650

[46] Zajkowska M, Zbucka-Krętowska M, Sidorkiewicz I, Lubowicka E, Gacuta E, Szmitkowski M, Lech Chrostek L, Ławicki S. Plasma levels and diagnostic utility of macrophage-colony stimulating factor, matrix metalloproteinase-9 and tissue inhibitor of metalloproteinase-1 as tumor markers in cervical cancer patients. Tumor Biol. 2018:1–9. Available from: 10.1177/1010428318790363. Accessed 30 Sept 2020. 10.1177/1010428318790363 30052166

[47] Lubowicka E, Zbucka-Kretowska M, Sidorkiewicz I, Zajkowska M, Ewa Gacuta E, Puchnarewicz A, Hrostek L, Szmitkowski M, Ławicki S. Diagnostic Power of Cytokine M-CSF, Metalloproteinase 2 (MMP-2) and Tissue Inhibitor-2 (TIMP-2) in Cervical Cancer Patients Based on ROC Analysis. Pathol Oncol Res. 2020;26:791–800. Available from: 10.1007/s12253-019-00626-z. Accessed 30 Sept 2020. 10.1007/s12253-019-00626-z PMC7242253 30820752

[48] Ruan F, Wang Y, Chai Y (2020). Diagnostic values of miR-21, miR-124, and M-CSF in patients with early cervical Cancer. Technol Cancer Res Treat.

[49] Będkowska GE, Ławicki S, Gacuta E, Pawłowski P, Szmitkowski M (2015). M-CSF in a new biomarker panel with HE4 and CA 125 in the diagnostics of epithelial ovarian cancer patients. J Ovarian Res.

[50] Ławicki S, Zajkowska M, Głażewska EK, Będkowska GE, Szmitkowski M. Plasma levels and diagnostic utility of VEG F, MMP-9, and TIMP-1 in the diagnosis of patients with breast cancer. Onco Targets Ther. 2016;9:911–9. Available from: 10.2147/OTT.399959. Accessed 13 Sept 2020. 10.2147/OTT.S99959 PMC4771393 26966379

[51] Cheng D, Liang B, Li Y. Serum Vascular Endothelial Growth Factor (VEGF-C) as a Diagnostic and Prognostic Marker in Patients with Ovarian Cancer. PLoS One. 2013;8(2):e55309. Available from: www.plosone.org. Accessed 30 Sept 2020. 10.1371/journal.pone.0055309 PMC3562180 23383322

[52] Urquidi V, Goodison S, Kim J, Chang M, Daib Y, Rosser CJ. VEGF, CA9 and Angiogenin as a Urinary Biomarker for Bladder Cancer Detection. Urology. 2012;79(5):1185.e1–6. Available from: 10.1016/j.urology. Accessed 30 Sept 2020. 10.1016/j.urology.2012.01.016 PMC3341520 22386755

[53] Jiang Y, Hu Z, Zuo Z, Li Y, Pu F, Biqiong Wang B, Tang Y, Guo Y, Tao H (2020). Identification of Circulating MicroRNAs as a Promising Diagnostic Biomarker for Cervical Intraepithelial Neoplasia and Early Cancer: A Meta-Analysis. Biomed Res Int.

[54] Derbie A, Mekonnen D, Woldeamanuel Y, Ostade XV, Tamrat Abebe T (2020). HPV E6/E7 mRNA test for the detection of high grade cervical intraepithelial neoplasia (CIN2+): a systematic review. Infect Agent Cancer.

[55] Tornesello ML, Faraonio R, Buonaguro L, Annunziata C, Starita N, Cerasuolo A, Francesca Pezzuto F, Tornesello AL, Buonaguro FM (2020). The Role of microRNAs, Long Non-coding RNAs, and Circular RNAs in Cervical Cancer. Front Oncol.

[56] Macedo AC, Gonçalves JCN, Bavaresco DV, Grande AJ, Chiaramonte Silva NC, Rosa MI (2019). Accuracy of mRNA HPV tests for triage of precursor lesions and cervical Cancer: a systematic review and meta-analysis. J Oncol.

[57] Kim HJ, Cho H, Choi CH, Joon-Yong Chung JY, Hewitt SM (2015). MicroRNA as biomarkers for cervical Cancer. J Gynecol Obstetr.

[58] Li J, Hu L, Tian C, Lu F, Wu J, Liu L (2015). microRNA-150 promotes cervical cancer cell growth and survival by targeting FOXO4. BMC Mol Biol.

[59] Wang X, Yu M, Zhao K, He M, Ge W, Sun Y, Wang Y, Sun H, Hu Y. Upregulation of MiR-205 under hypoxia promotes epithelial–mesenchymal transition by targeting ASPP2. Cell Death and Disease. 2016;7:e2517. Available from: 10.1038/cddis.2016.412. Accessed 30 Sept 2020. 10.1038/cddis.2016.412 PMC5261019 27929537

[60] Condrat CE, Thompson DC, Barbu MG, Bugnar OL, Boboc A, Cretoiu D (2020). miRNAs as Biomarkers in Disease: Latest Findings Regarding Their Role in Diagnosis and Prognosis. Cells.

[61] Chung D, Park S, Eom K, Park KH, Less HLS (2018). Feasibility of miR-9, miR-21, and miR-155 as alternative biomarkers in both HPV-positive and HPV-negative cervical cancers. J Clin Oncol.

[62] Sun H, Shen K, Cao D (2019). Progress in immunocytochemical staining for cervical cancer screening. Cancer Manag Res.

[63] Ngugi CW, Schmidt D, Wanyoro K, Boga H, Wanzala P, Muigai A, Mbithi J, von Knebel MD, Reuschenbach M. p16INK4a/Ki-67 dual stain cytology for cervical cancer screening in Thika district, Kenya. Infect Agents Cancer. 2015;10:25. Available from: 10.1186/s13027-015-0020-2. Accessed 30 Sept 2020. 10.1186/s13027-015-0020-2 PMC4531480 26265934

[64] Orang’o EO, Were E, Rode O, Muthoka K, Byczkowski M, Sartor H, Broeck DV, Schmidt D, Reuschenbach M, Doeberitz MVK, Bussmann M. Novel concepts in cervical cancer screening: a comparison of VIA, HPV DNA test and p16INK4a/Ki-67 dual stain cytology in Western Kenya. Infect Agents Cancer. 2020;15:57. Available from: 10.1186/s13027-020-00323-6. Accessed 18 Oct 2020. 10.1186/s13027-020-00323-6 PMC7531147 33024449

[65] Wentzensen N, Fetterman B, Tokugawa D, Schiffman M, Castle PE. Interobserver Reproducibility and Accuracy of p16/Ki-67 Dual-Stain Cytology in Cervical Cancer Screening. Cancer Cytopathol. 2014. Available from: 10.1002/cncy.21473. wileyonlinelibrary.com. Accessed 30 Sept 2020. 10.1002/cncy.21473 25132656

[66] Prevodnik VK, Jerman T, Nolde N, Fokter AR, Jezeršek S, Marinšek ZP, Klopčič U, Čelik SH. Interobserver variability and accuracy of p16/Ki-67 dual immunocytochemical staining on conventional cervical smears. Diagn Pathol. 2019;14:48. Available from: 10.1186/s13000-019-082. Accessed 30 Sept 2020. 10.1186/s13000-019-0821-5 PMC6533697 31122253

[67] Prevodnik VK, Marinsek ZP, Zalar J, Rozina H, Kotnik N, Jerman T, Jerneja Varl J, Ivanus U (2020). Evaluation of the training program for p16/ Ki-67 dual immunocytochemical staining interpretation for laboratory staff without experience in cervical cytology and immunocytochemistry. Radiol Oncol.

[68] Patel K, Strother RMN. Development of immunohistochemistry services for cancer care in western Kenya: implications for low- and middle-income countries. Afr J Lab Med. 2016;5:1. Available from: 10.4102/ajlm.v5i1.187. Accessed 30 Sept 2020. 10.4102/ajlm.v5i1.187 PMC5436389 28879100

[69] Termrungruanglert W, Khemapech N, Tantitamit T, Havanond P. Cost effectiveness analysis of HPV primary screening and dual stain cytology triage compared with cervical cytology. J Gynecol Oncol. 2019;2005(2005–0380·eISSN):2005–0399. Available from: 10.3802/jgo.2019.30.e17. Accessed 30 Sept 2020. 10.3802/jgo.2019.30.e17 PMC6393632 30740950

[70] Xu W, Xu M, Wang L, Zhou W, Xiang R, Shi Y, Zhang Y, Piao Y (2019). Integrative analysis of DNA methylation and gene expression identified cervical cancer-specific diagnostic biomarkers. Signal Transduct Target Ther.

[71] Yoon SM, Shin KH, Kim JY, Seo SS, Park SY, Moon SH, Cho KH (2010). Use of serum squamous cell carcinoma antigen for follow-up monitoring of cervical cancer patients who were treated by concurrent chemoradiotherapy. Radiat Oncol.

[72] Porika M, Vemunoori AK, Tippani R, Mohammad A, Bollam SR, Abbagani S (2010). Squamous cell carcinoma antigen and Cancer antigen 125 in southern Indian cervical Cancer patients. Asian Pac J Cancer Prev.

[73] Fu J, Wang W, Wang Y, Liu C, Wang P (2019). The role of squamous cell carcinoma antigen (SCC Ag) in outcome prediction after concurrent chemoradiotherapy and treatment decisions for patients with cervical cancer. Radiat Oncol.

[74] Guo H, Bi X, Lei T, Lv X, Yao G, Chen Y, Liu C (2020). Preoperative SCC-Ag as a predictive marker for the use of adjuvant chemotherapy in cervical squamous cell carcinoma with intermediate-risk factors. BMC Cancer.

[75] Ceci C, Atzori MG, Lacal PM, Graziani G (2020). Role of VEGFs/VEGFR-1 Signaling and Its Inhibition in Modulating Tumor Invasion: Experimental Evidence in Dierent Metastatic Cancer Models. Int J Mol Sci.

[76] Zusterzeel PLM, Span PN, Dijksterhuis MGK, Thomas CMG, Sweep FCGJ, Massuger LFAG (2009). Serum vascular endothelial growth factor: a prognostic factor in cervical cancer. J Cancer Res Clin Oncol.

[77] Huchko MJ, Bukusi EA, Cohen CR (2011). Building capacity for cervical cancer screening in outpatient HIV clinics in the Nyanza province of western Kenya. Int J Gynecol Obstet.

[78] Khozaim K, Orang’o E, Christoffersen-Deb A, Itsura P, Oguda J, Muliro H, Ndiema J (2014). Successes and challenges of establishing a cervical cancer screening and treatment program in western Kenya. Int J Gynecol Obstet.

[79] Orang’o O, Liu T, Christoffersen-Deb A, Itsura P, Oguda J, Washington S, Chumba D, Pisharodi L, Cu-Uvin S, Rositch AF. Use of VIA, Pap smear, or HR-HPV testing in women living with HIV/AIDS for post-treatment cervical cancer screening: same tests, different priorities. PMC (Pubmed Central Archives); 2018. Available from: 10.1097/QAD.0000000000001327. PMC. https://www.ncbi.nlm.nih.gov/pmc/articles/PMC5420497/pdf/nihms831643.pdf. Accessed 30 Sept 2020.

